# Proteomics of muscle chronological ageing in post-menopausal women

**DOI:** 10.1186/1471-2164-15-1165

**Published:** 2014-12-23

**Authors:** Marine Gueugneau, Cécile Coudy-Gandilhon, Ophélie Gourbeyre, Christophe Chambon, Lydie Combaret, Cécile Polge, Daniel Taillandier, Didier Attaix, Bertrand Friguet, Andrea B Maier, Gillian Butler-Browne, Daniel Béchet

**Affiliations:** INRA, UMR 1019, Centre de Recherche en Nutrition Humaine, Université d’Auvergne, F-63122 Saint Genès Champanelle, France; Clermont Université, Université d’Auvergne, F-63000 Clermont-Ferrand, France; INRA, Plateforme d’Exploration du Métabolisme, Composante Protéique, F-63122 Saint Genès Champanelle, France; UPMC Université Paris 06, UMR 8256, Biological Adaptation and Ageing - IBPS, CNRS-UMR 8256, INSERM U1164, Sorbonne Universités, F-75005 Paris, France; Department of Internal Medicine, Section of Gerontology and Geriatrics, VU University Medical Center, Amsterdam, The Netherlands; Institut de Myologie, Centre de Recherches en Myologie UMR 974 76, INSERM U974, CNRS FRE 3617, Sorbonne Universités, UPMC Université Paris 06, F-75013 Paris, France; Pôle Endocrinologie, Diabétologie et Nutrition, Institut de Recherches Expérimentales et Cliniques, Université Catholique de Louvain, B-1200 Brussels, Belgium

**Keywords:** Human, Skeletal muscle, Ageing, Sarcopenia, Proteomics, Biomarkers

## Abstract

**Background:**

Muscle ageing contributes to both loss of functional autonomy and increased morbidity. Muscle atrophy accelerates after 50 years of age, but the mechanisms involved are complex and likely result from the alteration of a variety of interrelated functions. In order to better understand the molecular mechanisms underlying muscle chronological ageing in human, we have undertaken a top-down differential proteomic approach to identify novel biomarkers after the fifth decade of age.

**Results:**

Muscle samples were compared between adult (56 years) and old (78 years) post-menopausal women. In addition to total muscle extracts, low-ionic strength extracts were investigated to remove high abundance myofibrillar proteins and improve the detection of low abundance proteins. Two-dimensional gel electrophoreses with overlapping IPGs were used to improve the separation of muscle proteins. Overall, 1919 protein spots were matched between all individuals, 95 were differentially expressed and identified by mass spectrometry, and they corresponded to 67 different proteins. Our results suggested important modifications in cytosolic, mitochondrial and lipid energy metabolism, which may relate to dysfunctions in old muscle force generation. A fraction of the differentially expressed proteins were linked to the sarcomere and cytoskeleton (myosin light-chains, troponin T, ankyrin repeat domain-containing protein-2, vinculin, four and a half LIM domain protein-3), which may account for alterations in contractile properties. In line with muscle contraction, we also identified proteins related to calcium signal transduction (calsequestrin-1, sarcalumenin, myozenin-1, annexins). Muscle ageing was further characterized by the differential regulation of several proteins implicated in cytoprotection (catalase, peroxiredoxins), ion homeostasis (carbonic anhydrases, selenium-binding protein 1) and detoxification (aldo-keto reductases, aldehyde dehydrogenases). Notably, many of the differentially expressed proteins were central for proteostasis, including heat shock proteins and proteins involved in proteolysis (valosin-containing protein, proteasome subunit beta type-4, mitochondrial elongation factor-Tu).

**Conclusions:**

This study describes the most extensive proteomic analysis of muscle ageing in humans, and identified 34 new potential biomarkers. None of them were previously recognized as differentially expressed in old muscles, and each may represent a novel starting point to elucidate the mechanisms of muscle chronological ageing in humans.

## Background

Ageing affects most tissues and physiologic functions, and one of the most affected organs is the skeletal muscle. The progressive decline in muscle mass and function due to ageing, which is also referred to as sarcopenia [1], contributes to both loss of autonomy [2], increased prevalence of falls, decreased resistance to metabolic aggression that increases morbidity [3] and mortality [4]. Numerous theories have been proposed to explain muscle ageing. Obviously, this is a multifactorial phenomenon which implicates intrinsic factors such as perturbations in the endocrine system, neuronal remodelling, oxidative stress and deficiencies in muscle regeneration, extrinsic factors such as diet and exercise, and also probably other unknown mechanisms [5].

Age-related degenerative changes are reflected in alterations in muscle morphology, function, and biochemical properties. Muscle ageing is thus associated with muscle fiber atrophy [6, 7], reduced muscle regenerative capacity [8], and neuropathic processes leading to motor unit denervation [9]. Mitochondrial dysfunctions with decreased capacity of oxidative enzymes and a decline of mitochondrial ATP production may also be observed with ageing in skeletal muscles [10–12].

The overall functional, structural, and biochemical alterations in ageing muscle have been extensively studied, but the molecular mechanisms implicated remain to be specified. The differential expression profiles of mRNAs constitute a first essential level of information, but analyses of the expression profile of proteins in ageing are also required to understand the molecular mechanisms important for the muscle ageing process [13]. In fact, unlike the genome, the proteome varies in response to many physiological or pathological factors. In addition, the proteome is orders of magnitude more complex than the transcriptome due to post-translational modifications, protein oxidation or limited protein degradation [14].

Several studies have been conducted in rat muscle and proteomic profiling has demonstrated substantial alterations in muscle proteins involved in key metabolic pathways, myofibrillar remodelling, cytoskeleton organisation and mechanisms of cytoprotection and cytodetoxification [15–19]. However, few studies have been conducted with human muscle and results are contradictory. Gelfi et al. [20] have shown that several enzymes involved in oxidative metabolism, including ubiquinol-cytochrome c reductase or aspartate aminotransferase, were more abundant in elderly than in young people, while Short et al. [11] have demonstrated a decrease in these enzymes with ageing. In contrast, some results observed in rat muscle proteomic analyses were confirmed in human muscle, such as a decrease in enzymes involved in glycolytic metabolism and an increase in proteins involved in cytoprotection (carbonic anhydrase 3) and cytodetoxification (Hsp70) [20, 21].

Because epidemiological studies have indicated accelerated muscle wasting after the fifth decade with an approximately 2% reduction in muscle mass per year [22], we have undertaken a top-down differential proteomic approach to determine potential changes after the fifth decade of life and to identify novel biomarkers of muscle ageing. In a previous study, we used a shot-gun proteomics approach to identify 35 potential biomarkers [23]. Herein, we performed two dimensional gel electrophoreses (2DGE) using biopsies of *vastus lateralis* from mature adult (56 years) *vs.* old (78 years) women, and for better separation of proteins, two different strategies were conducted. Firstly, overlapping immobilized pH gradient (IPG) with three different pH ranges were used to improve muscle proteins separation, and secondly, we assessed low salt extracts to remove the high abundance myofibrillar proteins and improve the detection of low abundance proteins. The proteomic profiling of aged skeletal muscle fibers revealed a differential expression pattern of 67 potential biomarkers important for energy metabolism, contractile properties, calcium signaling, cytoprotection, regulation of protein misfolding, and proteolysis. The present study demonstrates that alterations of muscle function in elderly women are associated with severe perturbed protein expression patterns and identified 34 new potential biomarkers of sarcopenia that had not previously been described.

## Results and discussion

### Differentially expressed proteins during ageing

**Figure 1:**
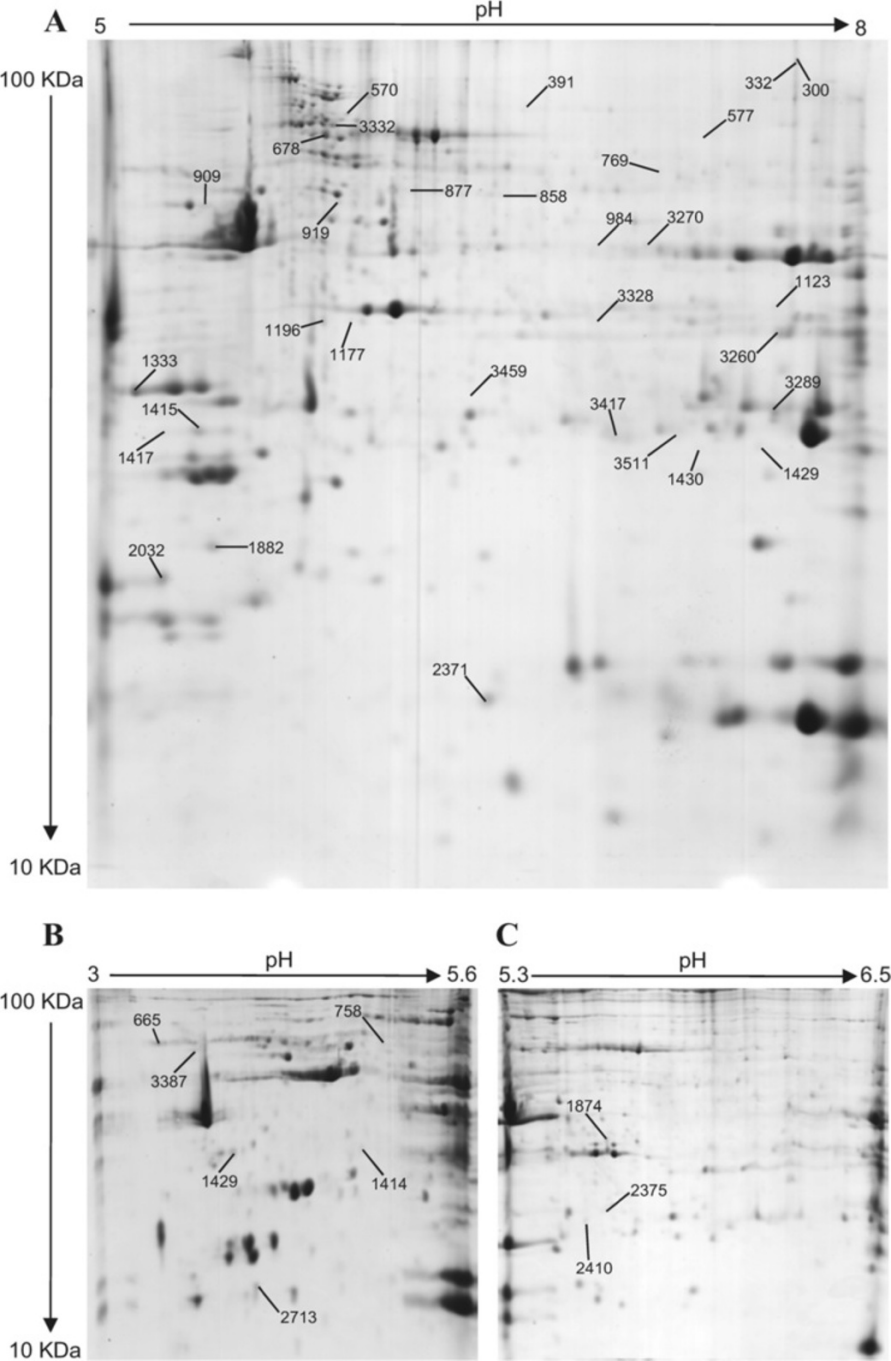
**Representative 2DGE image obtained from total protein extracts of human** ***vastus lateralis*** **skeletal muscle.** 2DGE was performed using a pH range of 5–8 **(A)**, 3–5.6 **(B)** or 5.6-6.5 **(C)** in first dimension and SDS-PAGE (11%T) in the second. Protein loading was 700 μg, and the gels were stained using colloidal Coomassie blue G-250. Differentially expressed and identified proteins are marked and spot numbers refer to Table 1.

In order to evaluate age-dependent alterations in the skeletal muscle proteome after the fifth decade of life, total muscle extracts from biopsies of mature adult (56 years) versus old (78 years) post-menopausal women were resolved by 2DGE. Gels with overlapping range of IPGs were used to improve the separation of total muscle extracts. 2DGE with medium range IPGs (pH 5–8) revealed 839 protein spots that were matched between all individuals. Among these protein spots, 56 were found to be differentially expressed between adult and old women, and 31 were identified by liquid chromatography coupled to tandem mass spectrometry (LC-MS/MS), corresponding to 27 different proteins (Figure 1A). 2DGE with acidic IPGs (pH 3.0-5.6) distinguished 202 matched protein spots, and among them 8 were differentially expressed and identified as 6 different proteins (Figure 1B). Narrow IPGs were also used to achieve optimal resolution in the pH 5.3-6.5 range, and this revealed a further 179 matched spots. Among these 179 spots, statistical analysis revealed 3 differentially expressed spots which were identified by LC-MS/MS as 3 different proteins (Figure 1C).

**Figure 2:**
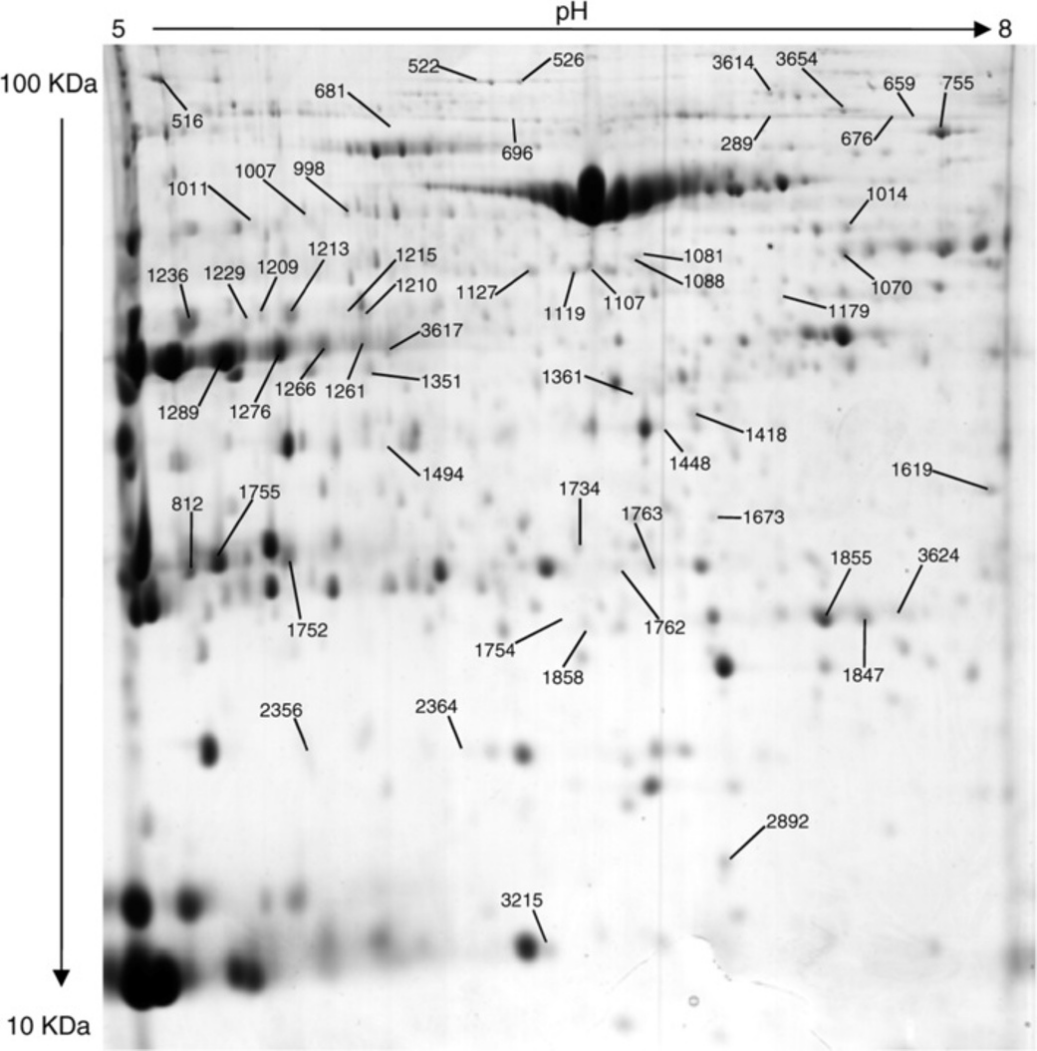
**Representative 2DGE image obtained from low ionic strength (LIS) extracts of human** ***vastus lateralis*** **skeletal muscle.** 2DGE was performed using a pH range of 5–8 in the first dimension and SDS-PAGE (11%T) in the second. Protein loading was 700 μg, and the gels were stained using colloidal Coomassie blue G-250. Differentially expressed and identified proteins are marked and spot numbers refer to Table 2.

Because myofibrillar proteins may hamper the detection of low abundance proteins, we also precipitated myofibrils at low ionic strength [24, 25], and focused on the soluble low ionic strength (LIS) extract. Thirty-six gels with medium range IPGs (pH 5–8) were used to analyze LIS extracts and 699 protein spots were matched between all individuals. Statistical analysis revealed that 86 spots were differentially expressed between adult and old women. Among them, 55 were identified by LC-MS/MS (Figure 2), and they corresponded to 37 different proteins. Targeting the LIS sub-proteome improved the 2DGE analysis of the muscle proteome, as most (32 out of 37) differentially expressed LIS proteins were not found in total muscle extracts.

Interestingly, shot-gun proteomics previously identified 35 potential biomarkers in the LIS sub-proteome [23]. It should be noted that, although shot-gun and 2DGE proteomics are based on different strategies (analyses of tryptic digests and intact protein isoforms, respectively), the two techniques are complementary, as only 6 common proteins were found in both investigations: titin (TTN), ankyrin repeat domain-containing protein 2 (ANKRD2), L-lactate dehydrogenase β (LDHB), furamate hydratase (FH), fatty acid binding protein 4 (FABP4) and carbonic anhydrase 3 (CA3). Overall in the present 2DGE study, 1919 protein spots were matched between all individuals, 95 were differentially expressed and identified, and they corresponded to 67 different proteins. Table 1 (for total muscle extract) and Table 2 (for LIS extract) summarize the main properties of the proteins differentially regulated in skeletal muscle between adult and old post-menopausal women. Absolute fold changes varied from 1.1 to 2.6. Even though some fold-changes appeared relatively small, there were parallel changes of several components in each pathway or biological process, suggesting some important coordinated and specific regulations.

**Table 1:** **Differentially expressed muscle proteins in total extract between adult (56 yr) and old (78 yr) post-menauposal women**

2DGE pH	Spot n°	Accession	Symbol	Protein name	Fold change	Age effect ( ***p*** value)	Mascot score	Sequence coverage (%)	Unique peptides	Main biological function
5-8	1882	P05976	MYL1	Myosin light chain 1/3	+1.4	0.020	693	41	5	Myofilaments and cytoskeleton
5-8	2032	P10916	MYL2	Myosin regulatory light chain 2	−1.7	0.012	4807	86	15	Myofilaments and cytoskeleton
5-8	1123	P45378	TNNT3	Troponin T, fast skeletal muscle	+1.9	0.033	288	24	4	Myofilaments and cytoskeleton
5-8	1333	P68133	ACTA1	Actin, alpha skeletal muscle (N-term fragment)	+1.7	0.042	999	23	4	Myofilaments and cytoskeleton
5-8	1415	P68133	ACTA1	Actin, alpha skeletal muscle (N-term fragment)	+1.3	0.034	847	29	4	Myofilaments and cytoskeleton
5-8	1417	P68133	ACTA1	Actin, alpha skeletal muscle (C-term fragment)	+1.3	0.041	350	19	4	Myofilaments and cytoskeleton
5-8	570	P12882	MYH1	Myosin-1 (C-term fragment)	+2.3	0.011	712	9	11	Myofilaments and cytoskeleton
3-5.6	665	P31415	CASQ1	Calsequestrin-1	+1.4	0.045	1146	22	9	Signal transduction
5-8	858	Q86TD4	SRL	Sarcalumenin (mature C-term)	−1.6	0.008	1606	44	17	Signal transduction
5-8	3260	Q9NP98	MYOZ1	Myozenin-1	+1.7	0.008	435	22	4	Signal transduction
3-5.6	758	P20073	ANXA7	Annexin A7	+1.8	0.029	954	15	6	Signal transduction, autophagy
5-8	300	P06732	CKM	Creatine kinase M-type (dimer)	−1.7	0.031	445	25	7	Energy metabolism
5-8	332	P06732	CKM	Creatine kinase M-type (dimer)	−1.7	0.048	737	39	11	Energy metabolism
5-8	391	P11217	PYGM	Glycogen phosphorylase, muscle form	−2.6	0.031	1516	39	23	Energy metabolism, glycogenolysis
5.3-6.5	1874	P21695	GPD1	Glycerol-3-phosphate dehydrogenase [NAD+], cytoplasmic	−1.4	0.040	3383	53	17	Energy metabolism, shuttle
5-8	3459	Q8N335	GPD1L	Glycerol-3-phosphate dehydrogenase 1-like protein	−1.4	0.022	1884	55	14	Energy metabolism, shuttle, hypoxia
5-8	3511	P60174	TPI1	Triosephosphate isomerase	+1.3	0.050	798	56	9	Energy metabolism, glycolysis, detoxification
5-8	1196	P11177	PDHB	Pyruvate dehydrogenase E1 component subunit beta	−1.3	0.043	2267	59	14	Energy metabolism, Krebs cycle
5-8	769	P09622	DLD	Dihydrolipoyl dehydrogenase, mitochondrial	−1.4	0.010	466	29	8	Energy metabolism, Krebs cycle
5-8	577	Q99798	ACO2	Aconitate hydratase, mitochondrial	−1.6	0.043	365	19	9	Energy metabolism, Krebs cycle
5-8	984	O75306	NDUFS2	NADH dehydrogenase [ubiquinone] iron-sulfur protein 2, mitochondrial	−1.6	0.005	1777	49	15	Energy metabolism, oxidative phosphorylation
5-8	3417	P47985	UQCRFS1	Cytochrome b-c1 complex subunit Rieske, mitochondrial	−1.3	0.031	1271	23	5	Energy metabolism, oxidative phosphorylation
3-5.6	2713	P20674	COX5A	Cytochrome c oxidase subunit 5A, mitochondrial	−1.5	0.015	779	48	6	Energy metabolism, oxidative phosphorylation
5-8	909	P06576	ATP5B	ATP synthase subunit beta	−1.4	0.023	2725	62	20	Energy metabolism, oxidative phosphorylation
5-8	2371	P05413	FABP3	Fatty acid-binding protein, heart	−1.4	0.004	2281	77	11	Energy metabolism, lipid transport
5-8	3328	P15121	AKR1B1	Aldose reductase	−1.5	0.016	323	31	5	Detoxification, cytoprotection
3-5.6	1414	P04792	HSPB1	Heat shock protein beta-1	+1.7	0.019	714	36	5	Detoxification, cytoprotection
5.3-6.5	2375	P04792	HSPB1	Heat shock protein beta-1	+1.3	0.026	484	41	6	Detoxification, cytoprotection
5-8	678	P08107	HSPA1A	Heat shock 70 kDa protein 1A/1B	+1.1	0.014	3630	52	14	Detoxification, cytoprotection
5-8	3332	P38646	HSPA9	Stress-70 protein, mitochondrial	+1.4	0.008	5401	45	22	Detoxification, cytoprotection
5-8	3289	P00918	CA2	Carbonic anhydrase 2	+1.3	0.047	1605	54	11	Detoxification, cytoprotection
5-8	877	Q13228	SELENBP1	Selenium-binding protein 1	−1.5	0.028	1677	56	19	Detoxification, cytoprotection
5.3-6.5	2410	P28070	PSMB4	Proteasome subunit beta type-4	−1.4	0.049	1692	33	5	Proteolysis
3-5.6	3387	P54725	RAD23A	UV excision repair protein RAD23 homolog A	−1.3	0.041	309	28	6	Proteolysis
5-8	3270	P49411	TUFM	Elongation factor Tu, mitochondrial	−1.4	0.001	1548	38	12	Protein synthesis, proteolysis
5-8	919	P02679	FGG	Fibrinogen gamma chain	−1.3	0.026	1023	59	16	Serum
5-8	1429	P30042	C21orf33	ES1 protein homolog, mitochondrial	−1.6	0.001	364	51	6	Miscellaneous
5-8	1430	P30042	C21orf33	ES1 protein homolog, mitochondrial	−1.7	0.002	118	18	3	Miscellaneous
5-8	1177	Q9H0P0	NT5C3	Cytosolic 5'-nucleotidase 3	−1.6	0.015	589	35	10	Miscellaneous
3-5.6	1429	Q9Y235	APOBEC2	Apolipoprotein B mRNA-editing enzyme, catalytic polypeptide-like 2	−1.2	0.018	1904	74	11	Miscellaneous

**Table 2:** **Differentially expressed muscle proteins in low ionic strength (LIS) extract between adult (56 yr) and old (78 yr) post-menauposal women**

Spot n°	Accession	Symbol	Protein name	Fold change	Age effect ( ***p*** value)	Mascot score	Sequence coverage (%)	Unique peptides	Main biological function
1734	E7ENC6	TTN	Titin (C-term fragment)	−1.4	0.032	4511	1	11	Myofilaments and cytoskeleton
1361	Q9GZV1	ANKRD2	Ankyrin repeat domain-containing protein 2	+1.8	0.033	1831	51	14	Myofilaments and cytoskeleton
522	P18206-2	VCL	Vinculin, isoform 1	+1.5	0.048	1457	21	17	Myofilaments and cytoskeleton
526	P18206-2	VCL	Vinculin, isoform 1	+1.6	0.026	1329	26	20	Myofilaments and cytoskeleton
1418	Q13643	FHL3	Four and a half LIM domains protein 3	−1.5	0.038	1046	42	8	Myofilaments and cytoskeleton
1179	P10644	PRKAR1A	cAMP-dependent protein kinase type I-alpha regulatory subunit	+1.6	0.021	903	37	11	Signal transduction
1494	P04083	ANXA1	Annexin A1	+1.3	0.049	4205	59	17	Signal transduction, dystrophies
1619	P08758	ANXA5	Annexin A5	+1.5	0.005	3264	64	16	Signal transduction, membrane repair
1673	P78417	GSTO1	Glutathione S-transferase omega-1	−1.5	0.049	674	44	7	Detoxification, signal transduction
1261	P06732	CKM	Creatine kinase M-type	−1.3	0.019	3974	45	15	Energy metabolism
1266	P06732	CKM	Creatine kinase M-type	−1.2	0.044	6576	50	16	Energy metabolism
1276	P06732	CKM	Creatine kinase M-type	−1.2	0.017	8749	57	17	Energy metabolism
1289	P06732	CKM	Creatine kinase M-type	−1.1	0.044	10394	52	17	Energy metabolism
3617	P06732	CKM	Creatine kinase M-type	−1.4	0.037	3259	43	13	Energy metabolism
516	P06732	CKM	Creatine kinase M-type (trimer)	−1.2	0.022	5360	54	16	Energy metabolism
289	P11217	PYGM	Glycogen phosphorylase, muscle form	+1.4	0.019	3091	48	28	Energy metabolism, glycogenolysis
659	F5GZH7	PYGM	Phosphorylase	+1.3	0.027	4260	49	31	Energy metabolism, glycogenolysis
676	F5GZH7	PYGM	Phosphorylase	+1.7	0.004	662	24	12	Energy metabolism, glycogenolysis
696	F5GZH7	PYGM	Phosphorylase	+1.5	0.004	917	23	15	Energy metabolism, glycogenolysis
998	P36871	PGM1	Phosphoglucomutase-1	−1.3	0.017	1772	33	14	Energy metabolism, glycogenolysis
1209	P13929	ENO3	Beta-enolase	−1.3	0.005	3382	41	12	Energy metabolism, glycolysis
1210	P13929	ENO1/3	Alpha/beta-enolase	−1.7	0.027	5218	53	12	Energy metabolism, glycolysis
1213	P13929	ENO3	Beta-enolase	−1.3	0.044	9731	62	20	Energy metabolism, glycolysis
1215	P13929	ENO3	Beta-enolase	−1.6	0.014	3548	49	13	Energy metabolism, glycolysis
1236	P13929	ENO3	Beta-enolase	−1.2	0.011	12846	62	22	Energy metabolism, glycolysis
1448	P07195	LDHB	L-lactate dehydrogenase B chain	−1.3	0.043	2156	46	11	Energy metabolism, glycolysis
1229	P07954-2	FH	Fumarate hydratase	−1.4	0.010	2328	44	12	Energy metabolism, Krebs cycle
3215	P15090	FABP4	Fatty acid-binding protein, adipocyte	+1.5	0.038	4748	70	10	Energy metabolism, lipid, ER stress
1007	P04040	CAT	Catalase	−1.3	0.026	654	21	9	Detoxification, cytoprotection
1754	E9PH29	PRDX3	Thioredoxin-dependent peroxide reductase, mitochondrial	+1.5	0.012	1802	47	9	Detoxification, cytoprotection
1858	Q99497	PARK7	Protein DJ-1	+1.2	0.043	334	42	6	Detoxification, cytoprotection
1107	P05091	ALDH2	Aldehyde dehydrogenase	+1.2	0.012	7985	48	19	Detoxification, cytoprotection
1351	P14550	AKR1A1	Alcohol dehydrogenase	+1.4	0.006	1387	46	13	Detoxification, cytoprotection
1011	P30038	ALDH4A1	Delta-1-pyrroline-5-carboxylate dehydrogenase	−1.2	0.027	1219	21	9	Amino acid metabolism
1762	P04792	HSPB1	Heat shock protein beta-1	+1.3	0.027	1453	51	7	Detoxification, cytoprotection
1763	P04792	HSPB1	Heat shock protein beta-1	+1.3	0.029	1286	51	8	Detoxification, cytoprotection
2356	E9PR44	HSPB5	Alpha-crystallin B chain	+1.5	0.038	1046	39	6	Detoxification, cytoprotection
2364	O14558	HSPB6	Heat shock protein beta-6	+1.2	0.043	597	71	4	Detoxification, cytoprotection
755	P08238	HSPC2/3	Heat shock protein HSP 90-alpha/beta	+1.9	0.035	3256	38	12	Detoxification, cytoprotection
1081	B3KQT9	PDIA3	Protein disulfide-isomerase A3	+1.4	0.012	965	36	15	Detoxification, cytoprotection
812	P00918	CA2	Carbonic anhydrase 2	+1.2	0.038	3352	72	15	Detoxification, cytoprotection
1752	P07451	CA3	Carbonic anhydrase 3	+1.2	0.004	3663	58	11	Detoxification, cytoprotection
1755	P07451	CA3	Carbonic anhydrase 3	+1.2	0.025	6098	72	14	Detoxification, cytoprotection
1119	Q13228	SELENBP1	Selenium-binding protein 1	+1.2	0.006	4100	70	23	Detoxification, cytoprotection
1127	Q13228	SELENBP1	Selenium-binding protein 1	+1.2	0.036	3543	63	21	Detoxification, cytoprotection
3614	P22314	UBA1	Ubiquitin-like modifier-activating enzyme 1	+1.8	0.033	7675	42	29	Proteolysis
3654	P55072	VCP	Transitional endoplasmic reticulum ATPase	+1.5	0.011	1920	39	23	Proteolysis
1088	P12955	PEPD	Xaa-Pro dipeptidase	+1.4	0.021	948	24	9	Proteolysis
1014	P01008	SERPINC1	Antithrombin-III	−1.2	0.014	3612	44	18	Proteolysis
1070	P02774	GC	Vitamin D-binding protein	−1.4	0.008	2704	40	14	Serum
2892	P02766	TTR	Transthyretin	−1.3	0.026	3695	65	8	Serum
1847	P02647	APOA1	Apolipoprotein A-I	−1.5	0.002	5385	55	15	Serum
1855	P02647	APOA1	Apolipoprotein A-I	−1.5	0.004	8189	54	15	Serum
3624	P02647	APOA1	Apolipoprotein A-I	−1.3	0.042	1347	41	8	Serum
681	P02787	TF	Serotransferrin	+1.7	0.047	1523	38	16	Serum

### Perturbations of the myofilament network and cytoskeleton with ageing

#### Sarcomeric proteins

Muscle contraction is generated by an interaction between the molecular motor myosin and filamentous actin. Myosin is a hexameric protein that consists of two heavy chain subunits, two alkali light chain subunits and two regulatory light chain subunits. Myosin light chains typically exhibit various isoforms, and in our study, muscle ageing was associated with higher level of one myosin light chain 1/3 skeletal muscle isoform (MYL1, spot 1882) and lower level of one myosin regulatory light chain 2 ventricular/cardiac muscle isoform (MYL2, spot 2032). Perturbations in myofibrillar contractile proteins were confirmed by the up-regulation of one isoform of fast troponin T (TNNT3, spot 1123) which is a major regulator of the thin filament. Troponin T directly interacts with key components in the thin filament regulatory system to mediate the activation and force development of actomyosin contractile units [26]. Age-related changes in fibers expressing various myosin light chain isoforms were previously described for MYL1, MYL2 and TNNT3 [15, 17, 20]; however, these changes were manifested differentially in distinct muscles.

Interestingly, the old skeletal muscle also exhibited altered levels of several fragments of sarcomeric proteins. Thus, there were higher levels of N-terminal (spots 1333 and 1415) and C-terminal (spot 1417) fragments of skeletal α-actin (ACTA1), and a higher level of a C-terminal fragment (spot 570) of myosin-1 (MYH1), the type IIX adult fast myosin heavy chain. The old muscle also exhibited lower level of a C-terminal fragment of TTN (LIS-spot 1734), when compared to adult muscle. Such fragments were not observed for non-sarcomeric proteins, and may indicate perturbations in sarcomeric proteolytic pathways.

A feature of human muscle ageing was also the increased expression of ankyrin repeat domain-containing protein 2 (ANKRD2, LIS-spot 1361). ANKRD2 is a member of the mechano-sensing proteins that link myofibrillar stress response to muscle gene expression [27, 28]. ANKRD2 interacts both with I-band sarcomeric proteins and with nuclear transcription factors [29]. This stretch-response protein is preferentially expressed in slow type-I fibers, and is induced by denervation, which is consistent with neuronal remodeling in ageing muscle [30].

Aging of skeletal muscle involves a decrease in both total number (hypoplasia) and size (atrophy) of muscle fibers. No consensus has been reached in the literature on whether hypoplasia is associated with a shift in fiber type distribution towards higher [31–33], lower [34, 35] or unaltered [12, 36, 37] percentages of type-I versus type-II fibers. However, previous studies based on myosin heavy chain (MYH) histochemistry agreed that atrophy mostly affects type-II fibers [12, 31, 32, 34, 38]. In our 2DGE analyses, there was no clear evidence for a fast-to-slow transition on the unique basis of the differential expression of myosin light chain and troponin isoforms.

#### Cytoskeletal proteins

The integrity of muscle fibers depends on cytoskeletal components, which align sarcomeres and anchor them across the sarcolemma to the basement membrane [39]. Two isoforms of vinculin (VCL, LIS-spots 522 and 526) were enhanced during ageing. VCL localizes to adhesion junctions and is a central component of muscle costameres [40, 41]. VCL is placed between the integrin-talin complex and the actin cytoskeleton and is a major candidate for transduction of force during the contractile cycle. Besides binding to actin, VCL interacts with signaling networks and is important for signal transduction between the extracellular matrix and the cytoskeleton [42].

Finally, the four and a half LIM domain protein 3 (FHL3, LIS-spot 1418), which is down-regulated in our analysis, is an adaptor protein with numerous interaction partners. In adult fibers, FHL3 interacts with cytoskeletal actin [43] and co-localizes with integrin receptors at the periphery of Z-discs [44]. By binding to integrin and actin, FHL3 might then directly link the cytoskeleton to the extracellular matrix. FHL3 also plays a role in myogenic progenitor cells (satellite cells), where it localizes to the nucleus and has been implicated in the regulation of proliferation [45] and differentiation [46].

**Figure 3:**
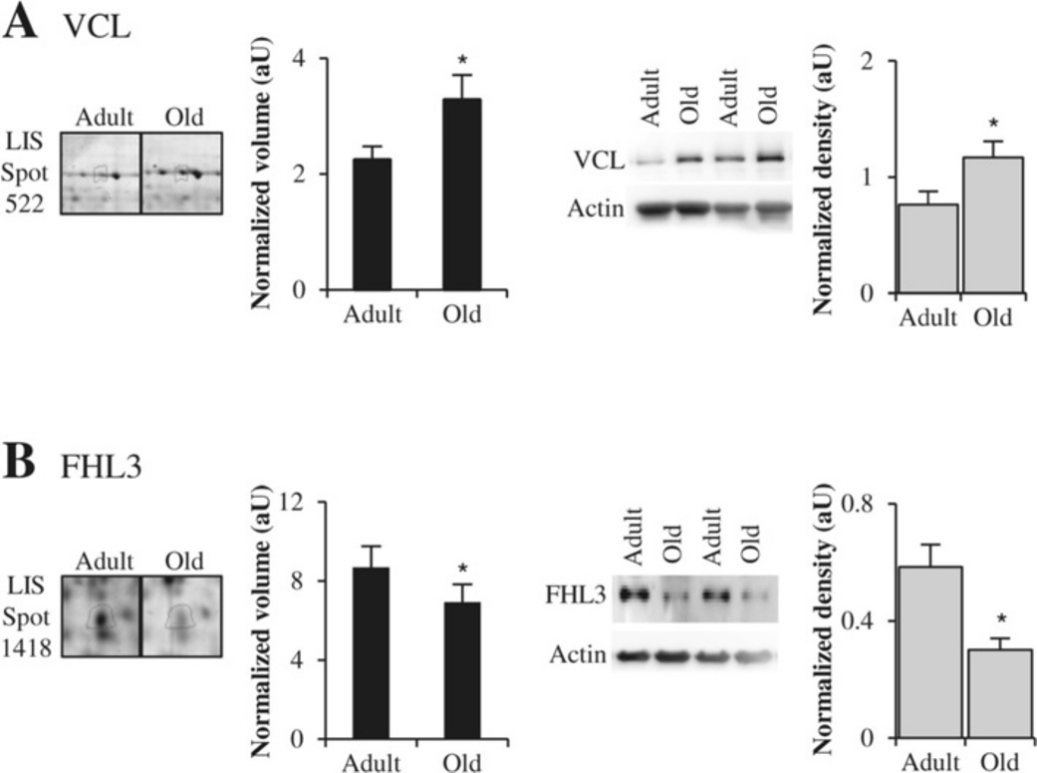
**Examples of differential expression of cytoskeletal proteins.** Representative sections of 2DGE images (left panel) and representative Western blots (right panel) for vinculin (VCL) **(A)** and four and a half LIM domains 3 (FHL3) **(B)**. In each panel, histograms represent normalized volume of protein spot (*n* = 6), and Western blot quantification (*n* = 7) for adult and old post-menopausal women. Results are indicated as means ± SE. *: *P* <0.05 indicates significant difference between adult and old women.

VCL and FHL3 have not been previously identified by proteomic surveys of human skeletal muscle ageing [11, 20, 21]. Shot-gun proteomics confirmed the up-regulation of ANKRD2 [23], and Western blotting experiments performed with total muscle extracts confirmed the differential expressions that we observed for VCL and FHL3 in LIS extracts (Figure 3A and B). In all, the modifications that we report for sarcomeric actomyosin and cytoskeletal proteins are most likely related to disorganization of myofibers in old muscles. Moreover, these changes suggest an influence of the ageing process on the maintenance of the proper organization of sarcolemma in regular structures that are closely linked to costameres, and on the response to extracellular signals. Thus, the age-related changes in sarcomeric and cytoskeletal proteins may result in alterations in contractile properties and contribute to the development of sarcopenia.

### Age-related changes in skeletal muscle affect signal transduction

In skeletal muscle, intracellular calcium (Ca^2+^) is an important secondary messenger for signal transduction and is essential for cellular processes such as excitation-contraction coupling. Action potentials elicit contractions by releasing Ca^2+^ from the sarcoplasmic reticulum (SR) *via* the ryanodine receptors (RyRs). RyRs are modulated directly or indirectly by various ions, small molecules and proteins, including calsequestrin. In this study, proteomics analysis of muscle ageing identified an up-regulation of calsequestrin-1 (CASQ1, spot 665), which is a major intra-SR Ca^2+^ buffer that regulates the activity of RyRs [47, 48].

RyR receptors have several potential phosphorylation sites in their cytoplasmic domains and protein kinase A (PKA) has been shown to phosphorylate RyRs. Our analysis shows an increased level of the PKA type Iα regulatory subunit (PRKAR1A, LIS-spot 1179) during ageing. Among other substrates PKA can phosphorylate RyR, and PKA-mediated phosphorylation of RyR may result in leaky RyR channels and impaired Ca^2+^ homeostasis [49, 50].

**Figure 4:**
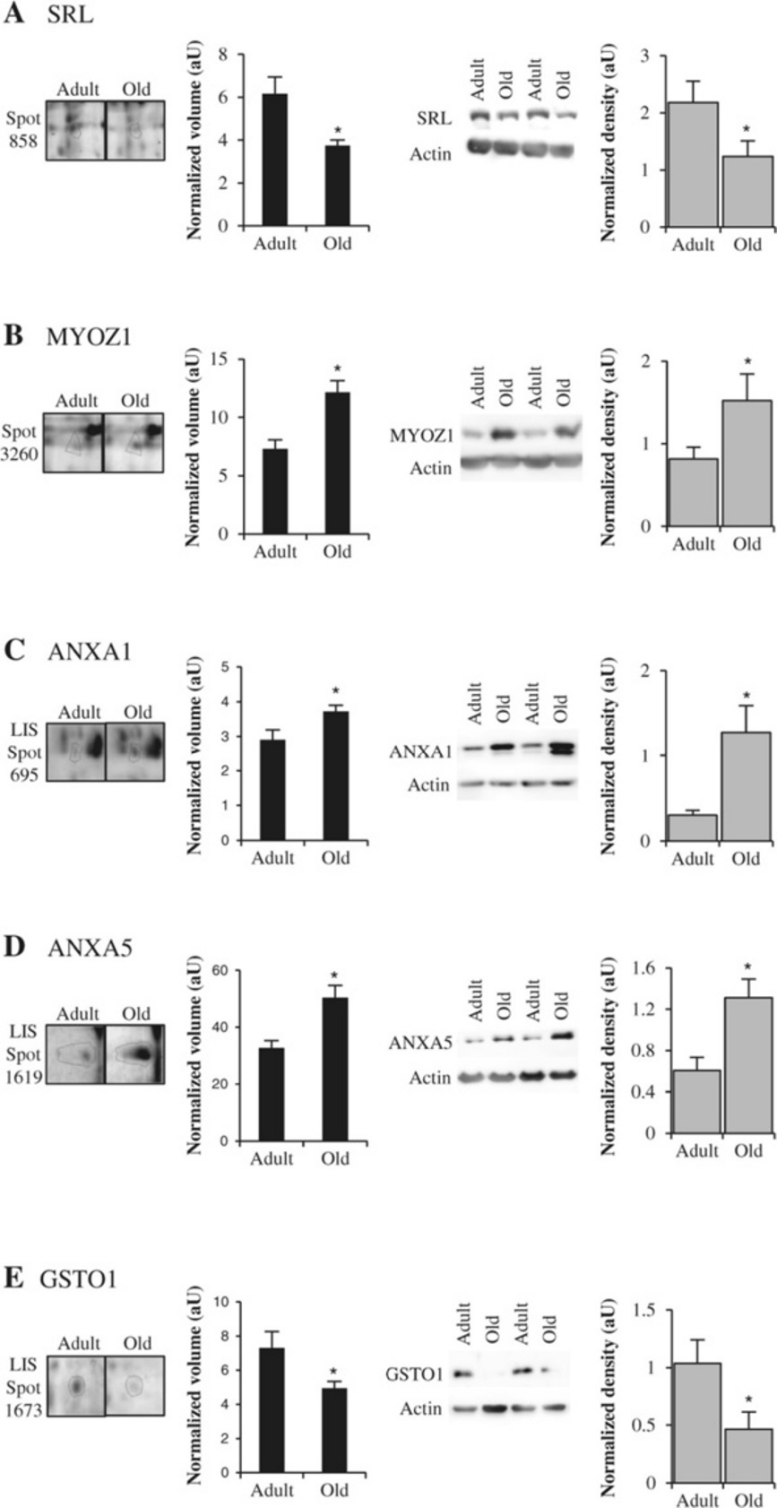
**Examples of differential expression of proteins implicated in signal transduction.** Representative sections of 2DGE images (left panel) and representative Western blots (right panel) for sarcalumenin (SRL) **(A)**, myozenin-1 (MYOZ1) **(B)**, annexin A1 (ANXA1) **(C)**, annexin A5 (ANXA5) **(D)** and glutathione S-transferase omega-1 (GSTO1) **(E)**. In each panel, histograms represent normalized volume of protein spot (*n* = 5–6), and Western blot quantification (*n* = 7) for adult and old post-menopausal women. Results are indicated as means ± *SE*. *: *P* <0.05 indicates significant difference between adult and old women.

After initiation of muscle contraction by increasing cytoplasmic Ca^2+^, Ca^2+^ is pumped back to the SR by sarcoplasmic reticulum Ca^2+^ ATPase (SERCA) leading to relaxation. Sarcalumenins (SRL) are major luminal glycoproteins that codistribute with SERCA and play a role in Ca^2+^ transport and sequestration [51, 52]. There are two SRL isoforms (160-kDa and 53-kDa) that are generated by alternative splicing [51]. In the present study, the 53-kDa isoform (spot 858) was found to be reduced in aged human muscle, and this reduction was confirmed by Western-blotting (Figure 4A). The age-related decrease of SRL is in agreement with previous studies in rat muscle indicating a shorter half-life of the 53-kDa isoform [53] and a lower level of the 160-kDa isoform [54].

We have also identified an up-regulation of myozenin-1 (MYOZ1, also termed calsarcin-2, spot 3260) which is expressed in fast-twitch fibers of skeletal muscle and modulates the function and substrate specificity of calcineurin, a Ca^2+^/calmodulin-dependent serine-threonine phosphatase that plays an important role in transducing calcium-dependent signals [55]. This increase in MYOZ1 was also confirmed by Western-blotting (Figure 4B).

In skeletal muscle, numerous proteins can bind Ca^2+^, and muscle ageing was further associated with higher level of three members of the annexin family which undergo Ca^2+^-dependent binding to the cellular membranes. We identified annexin A1 (ANXA1 or lipocortin I, LIS-spot 1494), A5 (ANXA5, LIS-spot 1619) and A7 (ANXA7 or Synexin, spot 758). Annexins have been involved in a broad range of molecular and cellular processes. Noteworthy ANXA1 may contribute to the regeneration of skeletal muscle tissue by modulating migration [56] and fusion [57] of satellite cells. ANXA1 is overexpressed in different muscular dystrophies [58] and can further participate in sarcolemmal and T-tubular repair processes [59]. ANXA5 also promotes membrane repair by self-assembling into two-dimensional arrays on membranes [60]. ANXA7 was originally described as a protein that provokes fusion of lipid vesicules [61]. More recently, ANXA7 was identified as an essential protein for autophagy induction by modulating the intracellular Ca^2+^ concentration [62]. Western-blotting experiments confirmed the overexpression of ANXA1 and ANXA5 (Figure 4C and D) in total extracts of old muscle, compared to adult muscle.

Glutathione S-transferase omega-1 (GSTO1) is distinguished from the other glutathione S-transferase family members by a different active center amino acid residue, which results in loss of prototypical glutathione conjugating activity [63]. Instead, human GSTO1 is reported to potentiate skeletal muscle ryanodine receptor (RyR1) [64]. The age-related down-regulation of GSTO1 (LIS-spot 1673) was confirmed by Western-Blot (Figure 4E), and may thereby be involved in the impairment of Ca^2+^ homeostasis.

Most of these signal transduction proteins (CASQ1, PRKAR1A, MYOZ1, ANXA1 and ANXA7) have never been reported in previous proteomic studies of muscle ageing. Overall, our findings suggest significant alterations in Ca^2+^ signaling which may be important for the age-related modifications in muscle contractile properties and may contribute to muscle weakness.

### Perturbations in the energy metabolism of old muscle

Disturbance in energy metabolism is another characteristic feature of old muscles. Decreased activities of glycolytic enzymes were previously reported in rat [15] and in human [20] skeletal muscles, whereas expression of mitochondrial enzymes was more controversial in the literature [11, 20]. Our study points to an age-associated decline in key enzymes of the glycolytic, Krebs cycle and oxidative phosphorylation pathways.

#### Cytoplasmic energy metabolism

In the current study, perturbations in the energy metabolism of old muscle were indicated by the down-regulation of five isoforms of monomeric creatine kinase (CKM) (LIS-spots 1261, 1266, 1276, 1289 and 3617). SDS-stable dimeric and trimeric forms of CKM were previously described in mice muscle [65], and we also observed age-related decreases in dimeric (spots 300 and 332) and trimeric (LIS-spot 516) forms of CKM. Creatine/phosphocreatine is central to maintain energetic homeostasis as it connects intracellular sites of energy demand with sites of ATP production. At these sites, CKM catalyses the transphosphorylation between phosphocreatine and ADP. In sarcomeric M-line, CKM interacts with myomesin and supplies ATP for the actomyosin contractile unit.

The first step of glycogenolysis pathway is the production of glucose-6-phosphate by glycogen phosphorylase (PYGM), which catalyzes the phosphorolytic cleavage of a glucosyl residue from the glycogen polymer. Four isoforms (LIS-spots 289, 659, 676 and 696) of PYGM increased, and one (spot 391) decreased with ageing. The resulting glucose 1-phosphate molecule is converted by phosphoglucomutase to glucose 6-phosphate. Proteomic analysis revealed a decreased expression of phosphoglucomutase-1 (PGM1, LIS-spot 998) in elderly women. Several glycolytic enzymes were also down-regulated with ageing. Enolase catalyzes the conversion of 2-phosphoglycerate to phosphoenolpyruvate. In the muscle of post-menopausal women, four isoforms of the muscle specific β-enolase (ENO3, LIS-spots 1209, 1213, 1215 and 1236) were selectively decreased during ageing. We also detected lower levels of LDHB (LIS-spot 1448) which catalyzes the inter-conversion of pyruvate (the final product of glycolysis) and lactate with concomitant inter-conversion of NADH and NAD^+^. Finally, down-regulations of glycerol-3-phosphate dehydrogenase (GPD1, spot 1874) and glycerol-3-phosphate dehydrogenase 1-like protein (GPD1L, spot 3459) were observed. Cytosolic GPD1, together with its mitochondrial isoform, constitute the GPD1 shuttle, which is essential for mitochondrial oxidation of glycolytic NADH. Although less active than its GPD1 counterpart, GPD1L exhibits dehydrogenase activity, and is also implicated in the regulation of hypoxia [66]. Age-related decline in cytoplasmic glycerol-3-phosphate dehydrogenase may indicate reduced mitochondrial oxidation of cytosolic NADH in old muscle.

Triosephosphate isomerase (TPI1, spot 3511), that catalyzes the isomerization of the dihydroxyacetone phosphate (DHAP) and D-glyceraldehyde 3-phosphate, was the only glycolytic enzyme more abundant in old muscle. TPI1 ensures that DHAP produced by aldolase is further metabolized by the glycolytic enzymes. Impairment of TPI1 can result in chemical conversion of DHAP into toxic methylglyoxal [67] promoting the formation of advanced glycation end-products. In the old muscle, an increased level of TPI1 may then represent a compensatory adaptation to avoid excessive formation of toxic products [68].

#### Mitochondrial energy metabolism

**Figure 5:**
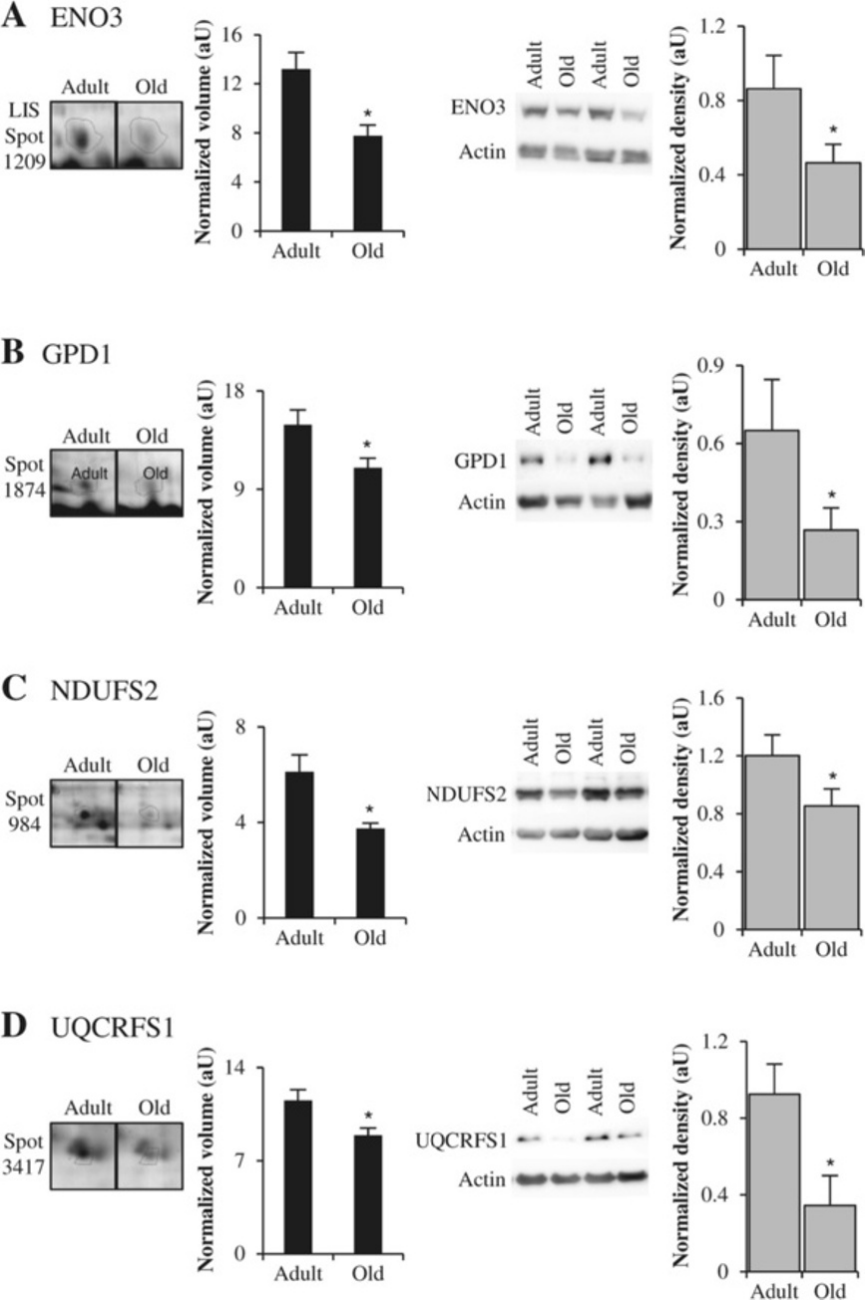
**Examples of differential expression of proteins implicated in energy metabolism.** Representative sections of 2DGE images (left panel) and representative Western blots (right panel) for β-enolase (ENO3) **(A)**, glycerol-3-phosphate dehydrogenase [NAD+] (GPD1) **(B)**, NADH dehydrogenase (ubiquinone) Fe-S protein 2 (NDUFS2) **(C)** and cytochrome b-c1 complex subunit Rieske (UQCRFS1) **(D)**. In each panel, histograms represent normalized volume of protein spot (*n* = 5–6), and Western blot quantification (*n* = 7) for adult and old post-menopausal women. Results are indicated as means ± *SE*. *: *P* <0.05 indicates significant difference between adult and old women.

Alterations in the mitochondrial Krebs cycle were revealed by the age-dependent reduction in two subunits of the pyruvate dehydrogenase, pyruvate dehydrogenase E1 component subunit β (PDHB, spot 1196) and dihydrolipoyl dehydrogenase (DLD, spot 769), and by the reduction in aconitate hydratase (ACO2, spot 577) and FH (LIS-spot 1229), two enzymes that catalyze the isomerization of citrate to isocitrate and the hydration of furamate to malate, respectively. Moreover, alterations in oxidative phosphorylation were also revealed by lower levels of three components of the respiratory chain, NADH dehydrogenase iron-sulfur protein 2 of complex I (NDUFS2, spot 984), cytochrome b-c1 complex subunit Rieske of complex III (UQCRFS1, spot 3417) and subunit 5A of cytochrome c oxidase of complex IV (COX5A, spot 2713). Ageing was further associated with lower level of ATP synthase subunit β (ATP5B, spot 909). Among these proteins implicated in energy metabolism, two (NDUFS2 and UQCRFS1) have never been reported in previous muscle ageing studies, and FH and ATP5B were similarly found in our shot-gun experiment [23]. Western blotting experiments confirmed alterations in glycolytic and oxidative metabolism, as we observed decreased expression of ENO3, GPD1, NDUFS2 and UQCRFS1, in total extracts of old muscle compared to adult muscle (Figure 5). Concomitant decreases of key enzymes of Krebs cycle and major complexes of oxidative phosphorylation provided evidences for alteration of mitochondrial metabolism in the old skeletal muscle.

#### Lipid metabolism

In addition to glucose, lipids are another source of energy in skeletal muscle. Several intracellular fatty acid-binding proteins (FABP) have been identified. They have important functions in the transport of intracellular fatty acids by increasing their solubility and have been shown to enhance the transport of fatty acids from the cell membrane to the site of oxidation, i.e. the mitochondria, and to the site of esterification into intramyocellular triglycerol [69]. Muscle ageing was associated with the down-regulation of heart FABP (FABP3, spot 2371), the major FABP in skeletal muscle, and the up-regulation of adipocyte FABP (FABP4, LIS-spot 3215). FABP3 plays an important, but merely permissive role in fatty acid uptake by skeletal muscles [70]. FABP4 is an adipocyte marker, but is also expressed in muscle fibers [69]. Therefore, the age-related up-regulation of FABP4 may relate to increased number of adipocytes, and/or to an increased expression in muscle fibers. Except in our shot-gun study [23], FABP4 was not noticed in previous reports on sarcopenia. Of note, the lipid chaperone FABP4 (also known as aP2) was also identified as a predominant positive regulator of toxic lipid-induced endoplasmic reticulum (ER) stress [71], and FABP4 up-regulation could suggest increased ER stress in old muscle.

Overall, our proteomic analyses therefore provided strong evidences for a decline in both glycolytic and mitochondrial energy metabolism in the old skeletal muscle. Declines in mitochondrial oxidative capacity were previously reported with advancing age [11], although physical activity, rather than chronological age, was also reported to be the primary determinant [72]. Because the activity score was significantly higher for the adult than for the old women group, future studies will be required to specify whether mitochondrial oxidative capacity still decreases in old subjects maintaining physical activity scores.

### Detoxification of cytotoxic products and cytoprotection in the old muscle

#### Protection against mitochondrial oxidative stress

Mitochondrial dysfunctions may lead to an excessive production of reactive oxygen species (ROS), and accumulating evidences suggest that oxidative stress underlies the ageing process in skeletal muscle [14]. The removal of H_2_O_2_ in cells is mediated by catalase (CAT), glutathione peroxidase and peroxiredoxin (PRDX) [73]. In the present study, ageing of human skeletal muscle was associated with lower levels of CAT (LIS-spot 1007) and higher levels of PRDX3 (thioredoxin-dependent peroxide reductase, LIS-spot 1754). Because CAT is localized preferentially in peroxisomes [74], its function is limited to the inactivation of H_2_O_2_ diffusing into these organelles. In contrast, PRDX3 is the only peroxiredoxin restricted to mitochondria, and high levels of PRDX3 may provide a primary line of defence against H_2_O_2_ over-produced by the respiratory chain in old muscle mitochondria. Neither CAT nor PRDX3 have previously been reported in proteomic analyses of muscle ageing.

A wide range of activities have been reported for protein DJ-1 (PARK7, LIS-spot 1858), however, there is consensus that PARK7 is responsive and protective against mitochondrial oxidative stress [75]. PARK7 is an atypical peroxiredoxin-like peroxidase [76]. During oxidative attack, PARK7 is relocalized to mitochondria, has a functional role in scavenging mitochondrial H_2_O_2_ and decreases mitochondrial fragmentation [77]. Therefore elevated levels of PARK7 in the old muscle may be important in regulating cellular antioxidant capacity.

#### Detoxification of cytotoxic products

Oxidative stress increases the production of cytotoxic aldehydes, which can react with cellular proteins, nucleic acids and cell membranes. Protection against reactive aldehydes is provided by several families of detoxification enzymes, including aldo-keto reductase (AKR) and aldehyde dehydrogenase (ALDH). Our proteomic analysis provided evidences for perturbed scavenging of reactive aldehyde products in the old muscle, as aldehyde dehydrogenase (ALDH2, LIS-spot 1107) and alcohol dehydrogenase (AKR1A1, LIS-spot 1351), were up-regulated, while aldose reductase (AKR1B1, spot 3328) and delta-1-pyrroline-5-carboxylate dehydrogenase (ALDH4A1, LIS-spot 1011) decreased with ageing. Similar regulations were previously reported during ageing in rat skeletal muscle for ALDH2 [15] and AKR1B1 [16, 78]. However, no previous study of muscle ageing has identified the differential expression of AKR1A1 and ALDH4A1.

**Figure 6:**
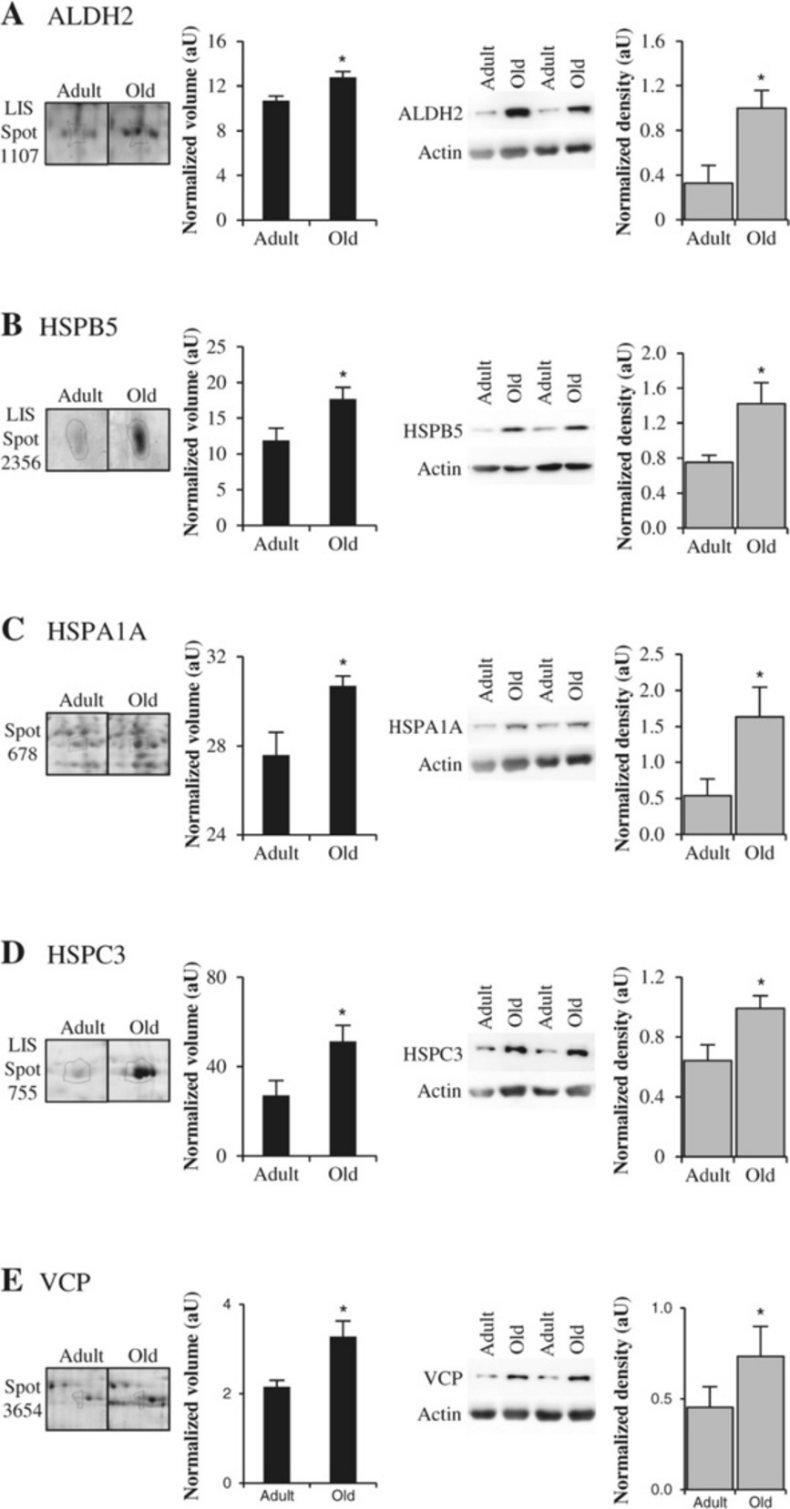
**Examples of differential expression of proteins implicated in cytoprotection, cytodetoxification or proteolysis.** Representative sections of 2DGE images (left panel) and representative Western blots (right panel) for aldehyde dehydrogenase (ALDH2) **(A)**, α-crystallin B chain (HSPB5) **(B)**, heat shock 70 kDa protein 1A/1B (HSPA1A) **(C)**, HSP 90-beta (HSPC3) **(D)**, and transitional endoplasmic reticulum ATPase (or valosin-containing protein, VCP) **(E)**. In each panel, histograms represent normalized volume of protein spot (*n* = 5–6), and Western blot quantification (*n* = 7) for adult and old post-menopausal women. Results are indicated as means ± *SE*. *: *P* <0.05 indicates significant difference between adult and old women.

The mitochondrial enzyme ALDH2 detoxifies aromatic and aliphatic aldehydes (including 4-hydroxy-2-nonenal), which are produced during oxidative stress as a result of lipid peroxidation [79]. The cytosolic oxidoreductase AKR1A1 has broad substrate specificity, and similarly catalyzes the reduction of aliphatic and aromatic aldehydes, ketones, and xenobiotics [80]. The up-regulations of ALDH2 and AKR1A1 thus suggest enhanced scavenging of reactive aldehyde products in the old skeletal muscle. Our Western blot analyses confirmed that muscle ageing is associated with higher levels of ALDH2 (Figure 6A).

While ALDH2 and AKR1A1 act principally as detoxification enzymes, AKR1B1 and ALDH4A1 have additional roles besides that of detoxification. Accumulating evidence attributes a significant role to AKR1B1 in transducing cytotoxic signals initiated by inflammatory cytokines [81]. AKR1B1 inhibitors have been reported to disrupt signalling cascades leading to NFkB activation [82], and reduced levels of AKR1B1 may therefore be important to limit activation of the NFkB pathway in the old muscle.

ALDH4A1 amino acid sequence diverges from the other ALDH [83] and this enzyme is mostly involved in metabolic regulation [84]. ALDH4A1 is metabolically important, since its substrate (glutamic y-semialdehyde) appears as a common intermediate in the degradative and biosynthetic pathways of the amino acids arginine, citrulline, ornithine and proline to and from glutamic acid. The down regulation of ALDH4A1 may thus indicate reduced intermediary metabolism in the old skeletal muscle.

#### Quality control of cellular proteins

To detect, refold, and eventually eliminate abnormal proteins, cells use quality control mechanisms that buffer protein homeostasis (proteostasis) against cellular stress. The proteomic analysis described here demonstrated the differential regulation of 9 spots identified as heat shock proteins (HSP). All were increased with ageing and they encompassed 3 HSP groups in human: HSPA, HSPB and HSPC [85].

The small heat shock proteins (HSPB) are ATP-independent chaperones. HSPBs prevent the aggregation of improperly folded or partially denatured proteins, and are involved in their transfer to the ATP-dependent chaperones or to the protein degradation processes such as proteasomes or autophagosomes [86]. Three HSPBs displayed higher levels in old women compared to adult women: heat shock protein beta-1 (HSPB1 or HSP27, spots 1414 and 2375, and LIS-spots 1762 and 1763), alpha-crystallin B chain (HSPB5 or CRYAB, LIS-spot 2356) and heat shock protein beta-6 (HSPB6 or HSP20, LIS-spot 2364). Similar up-regulation of HSPB1, HSPB5 and HSPB6 have been previously reported for rat muscle ageing [15, 17, 78, 87]. Our Western blot experiments performed with total muscle extracts confirmed that the old human muscle exhibited higher levels of HSPB5 than adult muscle (Figure 6B). In addition to chaperone activities, HSPB1 and HSPB5 have the ability to control the redox status, protect the actin cytoskeleton [88] and inhibit apoptotic cell death [89].

The ATP-dependent chaperones of the HSPA group (former HSP70) are essential for proteostasis as they contribute to the folding and assembly of nascent polypeptides, the transport of proteins across membranes, and the selection of misfolded proteins for degradation [90]. Muscle ageing was associated with the up-regulation of two HSPA proteins: the heat shock 70 kDa protein 1A/1B (HSPA1A or HSP70, spot 678), and the mitochondrial stress-70 protein (HSPA9 or GRP75, spot 3332). None of these HSPA have been previously reported in proteomic analyses of muscle ageing, and our Western blot experiments confirmed the age-related up-regulation of HSPA1A (Figure 6C). HSPA1A is the most abundant inducible cytoprotective HSP70 chaperone. Notably in rodents, overexpression of HSPA1A has been repeatedly reported to attenuate muscle atrophy induced by immobilization [91], lengthening contractions [92] or cryo-lesions [93]. It is therefore likely that the old human muscle may increase HSPA1A levels as a protection against muscle atrophy. HSPA9 is a central component of the mitochondrial protein import motor and it plays a key role in the folding of matrix-localized mitochondrial proteins. HSPA9 is the only known mitochondrial Hsp70 chaperone, and thus serves as a unique scavenger of toxic protein aggregates in human mitochondria [94].

One HSPC was further found to be up-regulated in these ageing muscles: the heat shock protein HSP 90 (isoform alpha or beta, LIS-spot 755). HSP 90, which has not been identified in previous muscle ageing studies, is another important chaperone that functions downstream of HSPA in the ATP-dependent folding and conformational regulation of many client proteins, including protein kinases, steroid receptors, endothelial NO synthase and transcription factors [95]. Most of the clients, with which HSP 90 interacts, are thus involved in signal transduction, making HSP 90 a critical factor in cell signalling. Western blot experiments confirmed the age-related up-regulation of HSP 90-beta (HSPC3, Figure 6D).

Finally and in addition to the HSP proteins, the chaperone operating in the endoplasmic reticulum (ER), protein disulfide-isomerase A3 (PDIA3, LIS-spot 1081), was also found to increase with human muscle ageing, and this observation is in agreement with previous studies in rat [15, 96]. PDIA3 is a multifunctional protein with thiol-protein disulphide oxidoreductase activity, which ensures proper folding of glycoproteins and assembly of major histocompatibility complex class I complex [97]. Outside the ER, PDIA3 also functions as a plasma membrane receptor for 1α,25-dihydroxy vitamin D3 [98], which might be important with regard to the implication of Vitamin D in ageing [99].

#### Cytoprotection in the old skeletal muscle

In agreement with a previous study in rat [78], higher levels of two isoforms of carbonic anhydrase 2 (CA2, spot 3289 and LIS-spot 812) were identified in senescent human muscle. No consensus has been reached in the literature about CA3, as both increased [16, 21] and decreased levels [11, 17] were observed in old muscles. Our analyses of muscle ageing in women indicated an overexpression of 2 isoforms of CA3 (LIS-spots 1752 and 1755) and this was confirmed by our shot-gun study [23]. The various isoforms of CAs play a crucial role in CO_2_-removal and CO_2_-provision for metabolic processes, and are central for the acid–base balance and the regulatory processes of ion homeostasis. Numerous proteins are involved in ion homeostasis, including the selenium-binding protein 1 (SELENBP1) of which two isoforms were increased (LIS-spots 1119 and 1127) and one isoform was decreased (spot 877) in ageing.

### Proteolytic systems

Muscle proteins are continuously turning over, and cells contain multiple proteolytic systems to carry out the degradation process. Several components of the major proteolytic systems, i.e. ubiquitin-proteasome [100] and lysosomal autophagy [101, 102], were in the present study differentially regulated with ageing in human skeletal muscle. The old muscle thus exhibited higher levels of the ubiquitin-like modifier activating enzyme 1 (UBA1, LIS-spot 3614), and transitional endoplasmic reticulum ATPase (or valosin-containing protein, VCP, LIS-spot 3654), while the proteasome subunit beta type-4 (PSMB4, spot 2410), UV excision repair protein RAD23 homolog A (RAD23A, spot 3387) and mitochondrial elongation factor Tu (TUFM, spot 3270) were down-regulated during ageing.

The VCP ATPase binds multiple ubiquitin ligases and ubiquitinated proteins and triggers extraction of client proteins from complexes or cellular surfaces, often to facilitate degradation by the proteasome [103]. VCP is thus central for the extraction and degradation of misfolded endoplasmic reticulum-associated (ERAD) [104] and mitochondrial membrane proteins [105]. VCP is also involved in a wide variety of cellular processes, such as DNA repair [106], myofibril biogenesis [107], membrane fusion [108], autophagosome maturation [109] and mitophagy [110]. The up-regulation of VCP in skeletal muscle of old women was confirmed by Western-blot analysis (Figure 6E), and is in agreement with previous observations in rat muscle [111]. The scaffold protein RAD23A, which was originally identified as an important factor involved in the recognition of DNA lesions [112], also serves as an ubiquitin receptor and plays a central role in targeting polyubiquitynated proteins, including ERAD substrates, for proteasomal degradation [113]. TUFM is important to deliver aminoacyl-tRNA to mitochondrial ribosome, and also promotes autophagy by interacting with autophagy-related proteins ATG12–ATG5 and ATG16L1 [114].

In addition to intracellular proteolysis, proteomics analysis of muscle ageing identified an up-regulation of the Xaa-Pro dipeptidase (PEPD, LIS-spot 1088), an extracellular proteinase which specifically splits iminodipeptides with C-terminal proline or hydroxyproline. Because of the high level of iminoacids in collagen, PEPD seems to be important for extracellular matrix remodeling [115]. At the extracellular level, we also found that muscle ageing was associated with lower level of anti-thrombin III (SERPINC1, LIS-spot 1014), which belongs to the family of serine protease inhibitors.

With the exception of VCP [111], none of these potential proteolysis biomarkers has previously been mentioned in any proteomic study on muscle ageing.

### Serum and transport proteins

Several spots were identified as differentially expressed plasma transport proteins. A number of these proteins were found decreased in aged muscle, including vitamin D binding protein (GC, LIS-spot 1070), transthyretin (TTR, LIS-spot 2892) and apolipoprotein A-I (APOA1, LIS-spots 1847, 1855 and 3624). Vitamin D binding protein is a major plasma transport protein for vitamin D, while TTR transports thyroxine (T4) and retinol (vitamin A) through the association with retinol-binding protein. As a major component of the high density lipoprotein complex, APOA1 helps to clear fats, including cholesterol, from peripheral tissues. APOA1 is also partly localized on skeletal muscle lipid droplets [116]. In contrast, serotransferrin (TF, LIS-spot 681), which controls level of iron and oxidative stress by increasing iron uptake, was up-regulated in aged muscle. Another serum protein, fibrinogen gamma chain (FGG, spot 919), was decreased during ageing. Altered levels of serum proteins may be related to impaired blood-flow distribution that has been reported in aged muscle fibers [117].

### Miscellaneous

Two isoforms of the ES1 protein homolog (C21orf33, spots 1429, 1430) also decreased in aged muscle. C21orf33 (also known as KNP1) is ubiquitously expressed but strongly so in heart and skeletal muscle and potential mitochondrial targeting signals were found in this protein [118], but its actual physiologic function is not known. Cytosolic 5'-nucleotidase 3 (NT5C3A, spot 1177) mainly catalyzes the dephosphorylation of pyrimidine nucleoside monophosphates, and therefore plays an important role in both endogenous nucleoside and nucleotide pool balance [119]. The age-related down-regulation of NT5C3A (−1.6 fold) that we observed in senescent women has not been previously reported in the literature. Finally, spot 1429 decreased 1.2-fold and is identified as apolipoprotein B mRNA-editing enzyme, catalytic polypeptide-like 2 (APOBEC2) that belongs to the cytidine deaminase superfamily and mediates editing of mRNA. Interestingly, APOBEC2 deficiency in mice has recently been associated with a shift to a slow-fiber type in muscle, and with diminished body mass and mild myopathy [120].

## Conclusions

**Figure 7:**
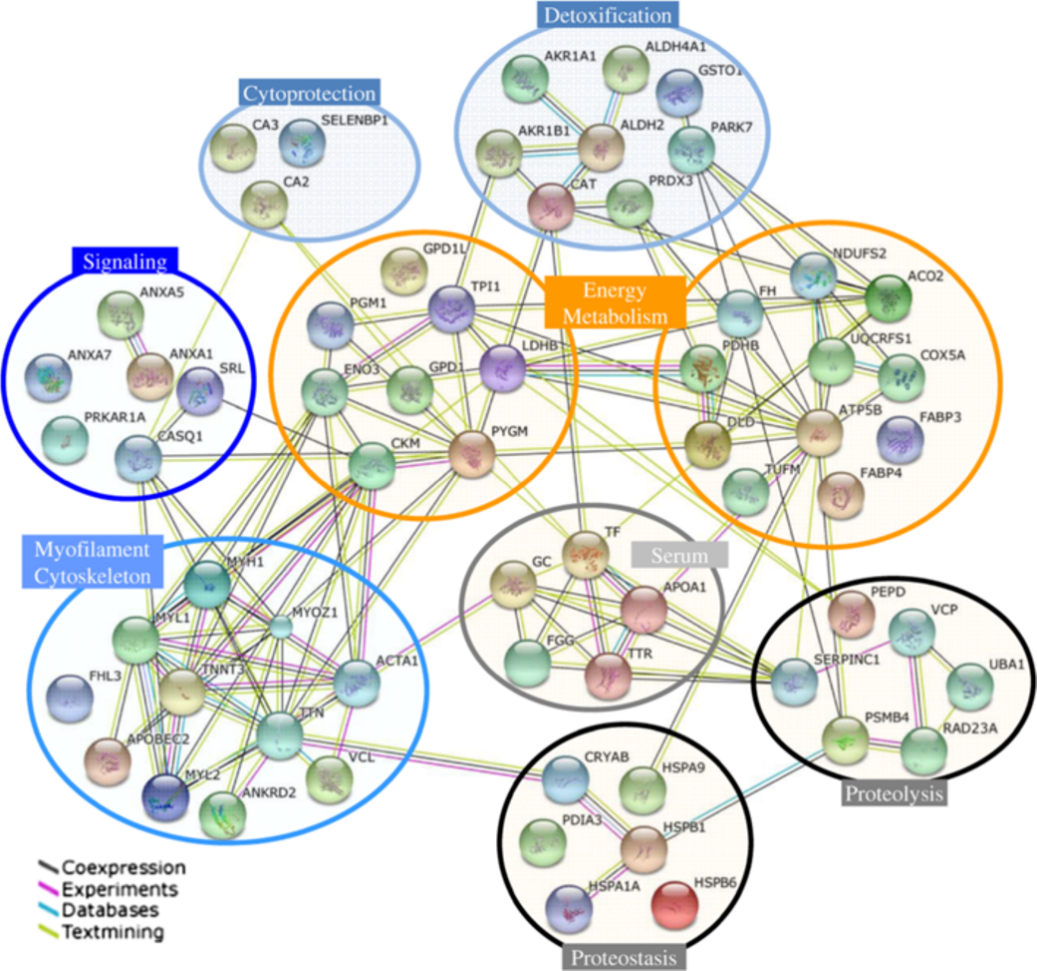
**STRING interaction network showing the association between differentially expressed proteins in old post-menopausal women compared to adult women.** The interaction map was generated using STRING [123] and default settings (Medium confidence of 0.4 and 4 criteria for linkage: co-expression, experimental evidences, existing databases and text mining). The proteins names used in this network are listed in Tables 1 and 2.

To conclude, our proteomics analyses has resulted in the identification of numerous proteins whose expression is dysregulated in old skeletal muscle. To our knowledge, this is the most extensive proteomic study of muscle ageing in humans. All these proteins have been classified into seven major groups, associated with myofilaments and cytoskeleton, energy metabolism, detoxification, cytoprotection, signal transduction, proteostasis and proteolysis, and Figure 7 summarizes the functional interaction networks linking the differentially expressed proteins. Many of the candidate proteins identified in this study by differential proteomics were previously unrecognized (34 out of 67) in previous ageing studies of skeletal muscle, in particular for proteins implicated in cytoprotection, signal transduction or proteolysis. By comparing adult and old post-menopausal women, we have identified a group of proteins in old skeletal muscle, which indicates potential mechanisms of ageing and may lead to the development of new biomarkers of sarcopenia and novel targets for therapeutic intervention.

## Methods

### Subjects

Subjects were admitted to the Leiden University Medical Center (Leiden, The Netherlands) and Rijnland Hospital (Leiderdorp, The Netherlands) between June 2010 and September 2012. Exclusion criteria consisted of previous knee or hip surgery (with the exception of arthroscopy), rheumatoid disease, diabetes mellitus, use of oral corticosteroids, and metastasized malignancy. Twenty four post-menopausal women undergoing elective hip surgery for hip arthrosis were selected in the present study and divided in two groups: adult women aged 56.4 ± 1.3 years (*n* = 11; age range 48–61 years) and old women aged 78.3 ± 0.5 years (*n* = 13; age range 76–82 years). We limited our study to post-menopausal women in order to avoid potential side effects of the menstrual hormone cycle. The study was approved by the medical ethical committees (P10.060 - HEALTH-2007-2.4.5-10: Understanding and combating age related muscle weakness “MYOAGE”) of the Leiden University Medical Center and Rijnland Hospital, and was performed in accordance with the principles of the revised Declaration of Helsinki. Written informed consent was obtained from all patients. The adult subjects were more active than elderly, but not involved in any specific training program. The activity score, based on self-report in a questionnaire, was significantly higher for adult than for old women (*P* <0.035). Adult women were cycling more than old women (83% and 36%, respectively), and adult women used less walking aid than old women (33% vs. 71%). The adult and old groups presented similar body weight (69.0 + 12.9 kg and 75.5 + 12.5 kg, respectively), height (168 + 8 cm and 166 + 7 cm, respectively) and body mass index. Muscle samples were obtained by surgical biopsy from the *vastus lateralis* muscle and were immediately frozen in liquid nitrogen and stored at −80°C until used.

### Reagents

Acrylamide, bisacrylamide and Hybond™-P membrane were purchased from Amersham Bioscience/GE Healthcare (Little Chalfont, UK). IPG buffers, 18 cm ReadyStrip IPG strips (pH 5–8), 18 cm ReadyStrip IPG strips (pH 3.0-5.6), 18 cm ReadyStrip IPG strips (pH 5.3-6.5), and Electrode Wicks were from Bio-Rad Laboratories (Marnes la Coquette, France), and orthophosphoric acid, ammonium sulphate and absolute ethanol were from VWR (Strasbourg, France). All other chemicals were from Sigma (L’Isle-d’Abeau Chesnes, France). Sequence grade-modified trypsin was purchased from Promega (Charbonnières-les-bains, France), and Luminata™ Western Horseradish peroxidase (HRP) substrate and ReBlot Plus Strong antibody stripping solution were from Millipore (Molsheim, France). For immunoblotting, the monoclonal antibodies against VCL and FHL3 were from Sigma. The anti-mouse IgG-HRP, anti-rabbit IgG-HRP, anti-SRL and anti-β-actin (ACTB) were from Santa-Cruz biotechnology (Heidelberg, Germany). The polyclonal antibodies against GPD1, MYOZ1, ANXA1, ANXA5, GSTO1, HSPB5, HSPC3, ALDH2, NDUFS2, UQCRFS1, ENO3, HSPA1A and VCP were purchased from Euromedex (Genetex, Souffelweyersheim, France).

### Protein extraction

Total muscle extracts and LIS extracts were prepared for each subject, and each individual was assessed separately. For total muscle extracts, biopsies of *vastus lateralis* muscle were from twelve post-menopausal women divided in two groups: adult control (*n* = 5) and aged (*n* = 7). Muscle aliquots were homogenized (40 mg/ml) in a solubilisation buffer containing 8.3 M urea, 2 M thiourea, 2% (w/v) CHAPS, 1% (v/v) dithiothreitol, and 2% (v/v) IPG buffer pH 3–10 using a TissueRuptor (Qiagen, Courtaboeuf, France), shaken for 30 min on ice and centrifuged for 30 min at 10,000 X *g*. The supernatants were aliquoted and stored at −20°C until analysis. Protein concentration, determined using the Bradford assay system (Bio-Rad), was 7.2 ± 1.1 mg/ml and 5.5 ± 1.2 mg/ml for the adult and old group, respectively.

LIS extracts were prepared from muscle biopsies of twelve post-menopausal women, either adult (*n* = 6) or aged (*n* = 6), according to Sayd et al. [121]. Briefly, frozen muscle was homogenized using a TissueRuptor in 40 mM Tris (pH 7.0), 2 mM EDTA, and protease inhibitor cocktail (Sigma). After centrifugation at 4°C for 10 min at 10,000 X *g*, the supernatant, referred to as LIS extract, was stored at −80°C. Protein concentration was 9.3 ± 2.2 mg/ml and 9.4 ± 1.6 mg/ml for the adult and old group, respectively.

### Two-dimensional gel electrophoresis

For total muscle extracts, 700 *μ*g protein were separated per gel using IPG strips of three different pH ranges (pH 3.0-5.6, pH 5.3-6.5 and pH 5–8) for each individual. For isoelectrofocusing, samples were diluted with rehydration buffer containing 8.3 M urea, 1 M thiourea, 2% (w/v) CHAPS, 0.28% (v/v) dithiothreitol, 2% (v/v) IPG buffer (pH 3–10), and 0.01% (w/v) Coomassie Brilliant blue R-250. The IPG strips were passively rehydrated with 330 μl of this solution for 16 h under mineral oil in the PROTEAN IEF Cell system (Bio-Rad) at 20°C, and actively rehydrated using Electrode Wicks loaded onto IPG strips for 6 h at 50 V. During active rehydration, Electrode Wicks were changed every 2 h. Isolectrofocusing was then performed at 0.05 mA per IPG strip at 50 V for 2 h, 200 V for 1 h, 500 V for 1 h, 1000 V for 2 h, 8000 V for 6 h and finally 8000 V to achieve 46,000 Vh.

For LIS extracts, gels were made in triplicate for each individual and 700 *μ*g protein was analyzed per gel. The IPG strips (pH 5–8) were passively and actively rehydrated as described above and isolectrofocusing was performed at 0.05 mA per IPG strip at 50 V for 1 h, 250 V for 1 h, 500 V for 1 h, 1000 V for 2 h, 1000 V for 1 h, 8000 V for 7 h and finally 8000 V to achieve 60,000 Vh.

The strips were then equilibrated twice for 15 min with gentle shaking in equilibration buffer containing 6 M urea, 50 mM Tris–HCl buffer (pH 8.8), 30% (v/v) glycerol, 2% (w/v) SDS. DTT (1% w/v) was added to the first, and iodoacetamide (5% w/v) to the second equilibration buffer. Separation of proteins, according to molecular weight, was carried out using a Protean Plus DodecaCell system (Bio-Rad) on homogenous 20 cm polyacrylamide gels (11% T, 2.6% C). The equilibrated strips were sealed to the top of the horizontal gel with agarose and subjected to 50 V for 1 h followed by 9 mA per gel until the blue dye reached the bottom of the gel.

### Visualization of proteins and image analysis

2DGE were fixed overnight in a solution containing 30% (v/v) ethanol and 2% (v/v) orthophosphoric acid, washed twice for 30 min in 2% (v/v) orthophosphoric acid and then transferred to a solution containing 18% (v/v) ethanol, 2% (v/v) orthophosphoric acid and 15% (v/v) ammonium sulphate for 30 min. The gels were stained for 72 h with 0.06% (w/v) Coomassie Blue G-250 added to this last solution. Gels were scanned using the ImageScanner and LabScan-v.5 software (Amersham Bioscience) and protein spots were analyzed and matched between all gels using Progenesis SameSpot software (Non Linear Dynamics, Newcastle upon Tyne, UK). Age effect was evaluated for normalized volumes of 2DGE spots using an unpaired Student’s *t* test procedure with significance set at *P* <0.05. The numbers of biological replicates (5–7) that we used in the present study is similar to those used in previous investigations [20, 21]. Throughout the manuscript, we qualified the differential spots as “potential” biomarkers, being aware that any list of candidate identified in a discovery stage must be validated in other large and independent cohort of subjects.

### Protein identification by mass spectrometry

Proteins with significant changed abundance were picked for tryptic digestion from gels. Excised protein spots from 2DGE were destained with 25 mM ammonium bicarbonate, 5% (v/v) acetonitrile for 30 min and twice in 25 mM ammonium bicarbonate, 50% (v/v) acetonitrile for 30 min each. Protein spots were then dehydrated using 100% acetonitrile for 10 min and dried in a vacuum SpeedVac. Proteins were digested overnight at 37°C using 12.5 ng/μl of sequence grade-modified trypsin (Promega) in 25 mM ammonium bicarbonate. Peptide extraction was optimised by adding 100% acetonitrile, followed by 15 min of sonication.

After concentration in the SpeedVac, the peptides mixtures were analyzed by nano-LC-MS/MS using an Ultimate 3000 system (Dionex, Voisins le Bretonneux, France) coupled to an LTQ-Velos mass spectrometer (Thermo Fisher Scientific, Bremen, Germany). Eight microliters of each peptide sample were loaded on a C18 pre-column (300-μm inner diameter × 5 mm; Dionex) at 20 μl/min in 5% acetonitrile, 0.05% trifluoroacetic acid. After 6 min of desalting, the pre-column was switched on line with the analytical C18 column (75-μm inner diameter × 15 cm; PepMap C18, Dionex) equilibrated in 96% solvent A (0.5% formic acid) and 4% solvent B (80% acetonitrile, 0.5% formic acid). Peptides were eluted using a 4-50% gradient of solvent B during 30 min at a 300 nl/min flow rate. The eluate was electrosprayed into the mass spectrometer through a nano-electrospray ion source. The LTQ-Velos was operated in a CID top 10 mode (1 full scan MS and the 10 major peaks in the full scan are selected for MS/MS). The Mascot Daemon software (version 2.3.2; Matrix Science, London, UK) was used to perform database searches, using the Thermo Proteome Discoverer v1.3 with default parameters to generate peak lists.

For protein identification, the UniP Human database (2012/11, 86675 seq) was used. Peptide mass tolerance was set to 1.5 Da and fragment mass tolerance was set to 0.8 Da. Two missed cleavages were allowed, and variable modifications were methionine oxidation and carbamidomethylation of cysteine. Identification results were imported and filtered by Proteome Discoverer. Protein identification was validated when at least three unique peptides originating from one protein showed significant identification Mascot scores (*P* <0.05) with False Discovery Rate (FDR < 5%) automatically calculated by Proteome Discoverer. When redundancy occurs, Proteome Discover uses principles of parsimony to give unambiguous identification of an isoform of a protein. This was not possible for only 2 spots (LIS spots 1210 and 755), for which we indicate the two isoforms in Table 2. In the present study we considered that proteins validated for the entire number of spots analysed were really present on the basis of Mascot score, percentage of sequence coverage of the entire protein, peptide spectrum matches, unique peptides related to each protein isoform/member, and MW agreements. The mass spectrometry proteomics data have been deposited to the ProteomeXchange Consortium [122] via the PRIDE partner repository with the dataset identifier PXD001527.

### Immunoblotting

For Western-Blot analysis, total protein extracts were resolved by sodium dodecyl sulfate polyacrylamide gel electrophoresis, electrotransferred to Hybon™-P membrane and probed with anti-VCL, anti-FHL3, anti-SRL, anti-MYOZ1, anti-ANXA1, anti-ANXA5, anti-GSTO1, anti-ENO3, anti-GPD1, anti-UQCRFS1, anti-NDUFS2, anti-ALDH2, anti-HSPB5, anti-HSPA1A, anti-HSPC3, or anti-VCP. Primary antibodies were resolved with corresponding horseradish peroxidase-linked goat anti-mouse or anti-rabbit secondary antibodies, and immunoreactive proteins were detected using enhanced chemiluminescence and a Charge Coupled Device camera (GBOX, Syngene, Cambridge, UK). Each blot was dehybridized using 1X ReBlot Plus Strong antibody stripping solution and probed with anti-ACTB for normalization. To determine the significance of ageing, a Student’s *t* test was used with significance set at *P* <0.05; results are expressed as the mean ± standard deviation.

### Functional correlation and pathway analysis

Pathway analysis was performed using the Search Tool for the Retrieval of INteracting Genes (String) 9.0 database (http://string-db.org) [123]. String analysis options were based on ‘evidence’ mode, we did not add or remove any protein partners, and we used clustering by K means to reveal sub-grouping within the network.

## Abbreviations

2DGE: Two-dimensional gel electrophoresis
ACO2: Aconitate hydratase
ACTA1: Skeletal α-actin
ACTB: β-actin
AKR: Aldo-keto reductase
AKR1A1: Alcohol dehydrogenase
AKR1B1: Aldose reductase
ALDH2: Aldehyde dehydrogenase
ALDH4A1: Delta-1-pyrroline-5-carboxylate dehydrogenase
ANKRD2: Ankyrin repeat domain-containing protein 2
ANXA1: Annexin A1
ANXA5: Annexin A5
ANXA7: Annexin A7
APOA1: Apolipoprotein A-I
APOBEC2: Apolipoprotein B mRNA-editing enzyme, catalytic polypeptide-like 2
ATG: autophagy-related protein
ATP5B: ATP synthase subunit β
C21orf33: ES1 protein homolog
CA: Carbonic anhydrase
CAP2: Adenylyl cyclase-associated protein 2
CAPNS1: Calpain small 1 regulatory subunit
CASQ1: Calsequestrin-1
CAT: Catalase
CKM: Creatine kinase
COX5A: Subunit 5A of cytochrome c oxidase
DHAP: Dihydroxyacetone phosphate
DLD: Dihydrolipoyl dehydrogenase
ENO3: Beta-enolase
ER: Endoplasmic reticulum
ERAD: Endoplasmic reticulum-associated
FABP: Fatty acid binding protein
FGG: Fibrinogen gamma chain
FH: Furamate hydratase
FHL3: Four and a half LIM domains protein 3
GC: Vitamin D binding protein
GPD1: Glycerol-3-phosphate dehydrogenase [NAD+]
GPD1L: Glycerol-3-phosphate dehydrogenase 1-like protein
GSTO1: Glutathione S-transferase omega-1
HRP: Horseradish peroxidase
HSP: Heat shock protein
HSPA1A: Heat shock 70 kDa protein 1A/1B
HSPA9: Mitochondrial stress-70 protein
HSPB1: Heat shock protein beta-1
HSPB5: α-crystallin B chain
HSPB6: Heat shock protein beta-6
HSPC3: Heat shock protein HSP 90-beta
LDHB: L-lactate dehydrogenase β
LIS: Low ionic strength
MYH: Myosin heavy chain
MYH1: Myosin-1
MYL1: Myosin light chain 1/3 skeletal muscle isoform
MYL2: Myosin regulatory light chain 2 ventricular/cardiac muscle isoform
MYOZ1: Myozenin 1
NDUFS2: NADH dehydrogenase (ubiquinone) Fe-S protein 2
NT5C3A: Cytosolic 5'-nucleotidase 3
PARK7: Parkinson disease 7, autosomal recessive early-onset
PDHB: Pyruvate dehydrogenase E1 component subunit β
PDIA3: Protein disulfide-isomerase A3
PEPD: Xaa-Pro dipeptidase
PGM1: Phosphoglucomutase-1
PKA: Protein kinase A
PRDX: Peroxiredoxin
PRDX3: Thioredoxin-dependent peroxide reductase
PRKAR1A: cAMP-dependent protein kinase type I-alpha regulatory subunit
PSMB4: Proteasome subunit beta type-4
PYGM: Glycogen phosphorylase
RAD23A: UV excision repair protein RAD23 homolog A
ROS: Reactive oxygen species
RyR: Ryanodine receptor
SELENBP1: Selenium-binding protein 1
SERCA: Sarcoplasmic reticulum Ca^2+^ ATPase
SERPINC1: Anti-thrombin III
SR: Sarcoplasmic reticulum
SRL: Sarcalumenin
TF: serotransferrin
TNNT3: Fast troponin T
TPI1: Triosephosphate isomerase
TTN: Titin
TTR: Transthyretin
TUFM: Mitochondrial elongation factor Tu
UBA1: Ubiquitin-like modifier activating enzyme 1
UQCRFS1: Cytochrome b-c1 complex subunit Rieske
VCL: Vinculin
VCP: Valosin-containing protein.
